# Characterization of intracranial compliance in healthy subjects using a noninvasive method - results from a multicenter prospective observational study

**DOI:** 10.1007/s10877-024-01191-w

**Published:** 2024-07-20

**Authors:** Gabriela Nagai Ocamoto, Lucas Normando da Silva, Camila da Silva Rocha Tomaz, Matheus Toshio Hisatugu, Gustavo Frigieri, Danilo Cardim, Roberta Lins Gonçalves, Thiago Luiz Russo, Robson Luis Oliveira de Amorim

**Affiliations:** 1 Braincare Desenvolvimento e Inovação Tecnológica S.A., Bruno Ruggiero Filho Avenue, 971, São Carlos, São Paulo, 13562-420 Brazil; 2 Health Sciences Postgraduation Program, Federal University of Amazonas, General Rodrigo Octavio Jordão Ramos Avenue, 1200, Manaus, Amazonas, 69067-005 Brazil; 3 Medical Investigation Laboratory 62, University of São Paulo School of Medicine, São Paulo, Brazil; 4 Department of Physical Therapy, Federal University of São Carlos, Washington Luís Road, km 235, São Carlos, São Paulo, 13565-905 Brazil

**Keywords:** Intracranial compliance, Intracranial pressure, Noninvasive monitoring, Children, Adults, Elderly

## Abstract

**Purpose:**

An FDA-approved non-invasive intracranial pressure (ICP) monitoring system enables the assessment of ICP waveforms by revealing and analyzing their morphological variations and parameters associated with intracranial compliance, such as the P2/P1 ratio and time-to-peak (TTP). The aim of this study is to characterize intracranial compliance in healthy volunteers across different age groups.

**Methods:**

Healthy participants, both sexes, aged from 9 to 74 years old were monitored for 5 min in the supine position at 0º. Age was stratified into 4 groups: children (≤ 7 years); young adults (18 ≤ age ≤ 44 years); middle-aged adults (45 ≤ age ≤ 64 years); older adults (≥ 65 years). The data obtained was the non-invasive ICP waveform, P2/P1 ratio and TTP.

**Results:**

From December 2020 to February 2023, 188 volunteers were assessed, of whom 104 were male, with a median (interquartile range) age of 41 (29–51), and a median (interquartile range) body mass index of 25.09 (22.57–28.04). Men exhibited lower values compared to women for both the P2/P1 ratio and TTP (*p* < 0.001). There was a relative rise in both P2/P1 and TTP as age increased (*p* < 0.001).

**Conclusions:**

The study revealed that the P2/P1 ratio and TTP are influenced by age and sex in healthy individuals, with men displaying lower values than women, and both ratios increasing with age. These findings suggest potential avenues for further research with larger and more diverse samples to establish reference values for comparison in various health conditions.

**Trial registration:**

Brazilian Registry of Clinical Trials (RBR-9nv2h42), retrospectively registered 05/24/2022. UTN: U1111-1266-8006.

**Supplementary Information:**

The online version contains supplementary material available at 10.1007/s10877-024-01191-w.

## Introduction

Intracranial compliance (ICC) and intracranial pressure (ICP) monitoring are of great interest to intensive care medicine, neurology, and related areas. ICP is a pivotal physiological parameter that reflects the dynamic equilibrium between the cranial contents and the cerebrospinal fluid circulation. Elevated ICP is a critical factor in numerous neurological conditions, including traumatic brain injury and stroke, which can lead to reduced cerebral perfusion and subsequent cellular damage [1]. However, the traditional methods for monitoring ICP are invasive, posing risks of infection and hemorrhage. Moreover, there is a growing body of evidence emphasizing the importance of analyzing the ICP waveform beyond the absolute number. None of the invasive methods commercially available analyze quantitatively the components of waveform such as the P2/P1 ratio, a known variable related to intracranial compliance [1–5].

Recent technological advancements have led to the development of non-invasive methods for ICP monitoring, such as the brain4care system, which measures micrometric pulsatile cranial expansions originated from ICP variations each cardiac cycle as an indicator of the ICP wave morphology [1, 5]. This FDA-approved non-invasive system monitors intracranial pulsation by analyzing the morphology of the intracranial pressure pulse variations (ICP waveform) and generating associated parameters, such as the P2/P1 ratio and time-to-peak (TTP).

This method has shown promise in detecting changes in ICC and conditions of intracranial hypertension based on ICP waveform morphology and associated parameters [6–10]. Despite these advancements, a significant gap exists in the literature: the lack of established reference values for ICP parameters in individuals without neurological conditions, which hinders the interpretation and broader application of these non-invasive techniques. The present study addresses these critical gaps by utilizing the brain4care system to establish reference values for ICP wave morphology in healthy individuals across a wide age range.

## Methods

### Study design and approval

This study was designed as a cross-sectional analysis and conducted in accordance with the STROBE guidelines. After approval of the ethics committee of the Federal University of São Carlos (CAAE number: 32338920.5.0000.5504 and IRB approval number: 5.570.885) and Federal University of Amazonas (CAAE number: 44977021.7.0000.5020 and IRB approval number: 4.722.851), a prospective observational study was conducted at the Department of Physical Therapy at the Federal University of São Carlos and at the Araújo Lima Outpatient Clinic - Getúlio Vargas University Hospital. The study was designed and performed in accordance with the guidelines of the Declaration of Helsinki and the requirements of the Brazilian Ethical Committee. Written informed consent was obtained from all participants before inclusion in the study. For children, the written informed consent was obtained from the parents or legal representatives. The trial was registered at the Brazilian Registry of Clinical Trials (RBR-9nv2h42), UTN: U1111-1266-8006.

### Setting and study population

The recruitment of participants was conducted through outreach efforts in the community, universities, gyms, schools, parks, and social media platforms in São Carlos, state of Sao Paulo and Manaus, state of Amazonas, Brazil from December 2020 to March 2023. Inclusion criteria were: aged 5 years or older, ability to provide informed consent or consent provided by parents or legal representatives, and ability to understand verbal commands. Exclusion criteria included: clinical signs of intracranial hypertension, abnormal neurological examination findings, history of previous brain surgery, uncontrolled systemic disease, current migraine or headache crisis, or pregnancy.

### Study procedures

During the recruitment process, potential participants were provided with an explanation of the study’s nature. A screening questionnaire was administered to determine if they met the inclusion criteria. Upon meeting these criteria, potential participants were invited to take part in the study and were asked to sign the informed consent form. Comprehensive information regarding the assessment process was then provided to each participant.

The study protocol involved a 1-day assessment conducted in a quiet and climate-controlled room, with the ambient temperature maintained around 20 °C. First, the written consent form was collected. Second, demographic data including age, sex, ethnicity, weight, height, waist and hip circumference, and health history (such as alcohol consumption, smoking, physical activity, anxiety, migraine, and use of medication for comorbidity control) were gathered. Body mass index (BMI) and waist-to-hip ratio were calculated from weight, height, and waist and hip circumference measurements. Third, preparation for intracranial compliance assessment was performed. Before initiating the ICC assessment, participants underwent a 5-minute rest period to stabilize vital signs—blood pressure, heart rate, respiratory rate, temperature, and oxygen saturation—which could be influenced by internal (healthcare service conditions) or external (environmental conditions) factors. Subsequently, vital signs were collected, and ICC was monitored continuously for 5 min. The P2/P1 ratio and TTP were generated minute by minute using a noninvasive system.

#### Demographic data and health history assessment

Demographic information, health history, and lifestyle habits data were collected using a structured questionnaire. The questionnaire was administered to volunteers, or answered by a legal representative when volunteers lacked understanding of the questions, such as in the case of illiterate children.

Questions regarding alcohol or tobacco consumption included inquiries about quantity and frequency of consumption. Alcohol consumption was categorized as non-drinker and drinker (volunteer consuming at least five drinks per month) [11]. Regarding smoking, a participant who reported consuming at least one cigarette per day for at least six months was considered a smoker [12].

The physical activity was assessed among participants based on the American College of Sports Medicine (ACSM) and the American Heart Association (AHA) recommendations [13, 14]. The assessment respected the amount of activity related to the participant’s age.

For sleep disorders, anxiety or history of migraine, the volunteer made a self-report based on sleep less or more than 7–9 h per day, presence or absence of symptoms of anxiety, and headache associated with nausea, vomiting or photophobia, respectively. Also, the questionnaire addressed the presence of any clinically diagnosed health conditions.

#### Intracranial compliance assessment

To assess the ICC, an FDA-approved noninvasive system was utilized (brain4care^®^ - Braincare Desenvolvimento e Inovação Tecnológica S.A., São Carlos, São Paulo, Brazil). The brain4care wireless system (BWS) comprises a wireless sensor that captures real-time data. These signals are transmitted via Bluetooth^®^ connection to a mobile application serving as a monitor. The collected data are then sent to the cloud, where analytical software processes it before transmitting it back to the mobile application [15]. The BWS provides an estimated P2/P1 ratio variation over time, an ICP waveform signal over time, and pulse morphology, averaged per minute, with the P2/P1 ratio and TTP.

An experienced professional positioned the sensor over the temporo-parietal region of the volunteer’s head. The volunteer maintained a neutral head alignment in a supine position. Signal recording commenced after completion of all preparation procedures, and data collection continued for 5 min. The supine position was chosen based on its common occurrence in daily hospital contexts, facilitating future comparisons under similar conditions. Volunteers were instructed to remain relaxed, avoiding head or body movements and refraining from speaking during the data collection period to minimize signal noise.

#### Collected data validation

**Fig. 1:**
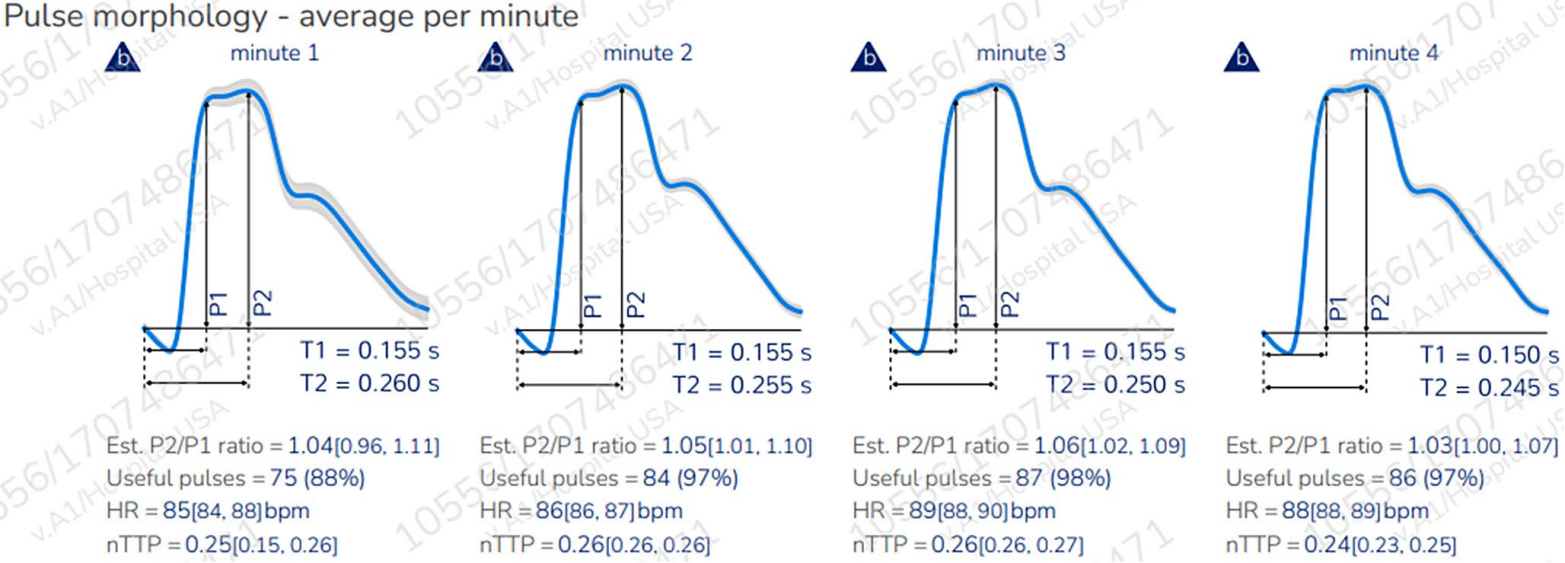
Illustration of ICP pulse morphology and parameters obtained by the brain4care system

The collected data underwent a rigorous curation process, carefully selected based on the quality of the processed signal to ensure the reliability and accuracy of subsequent analyses. Initially, the analytical software within the cloud performed several tasks including data parsing, detrending, signal validation, signal filtering, inversion verification, pulse identification, artifact removal, pulse alignment, pulse averaging, and pulse parameter calculation. Signal filtering involved a two-part algorithm: firstly, a 0–1 signal quality index based on spectral analysis was calculated over a one-minute downsampled and detrended data. The power spectral density derived from the signal is utilized to estimate the signal-to-noise ratio (SNR). The SNR is computed as the ratio of signal energy within the fundamental frequency and its first three harmonic frequencies, with a 0.2 Hz margin around each harmonic, relative to the total band spectrum spanning 0.1 Hz to 25 Hz. Signals exceeding an SNR threshold of 0.35 undergo mean pulse assessment, which serves as input for a quality classifier determining signal morphology acceptability. The resultant quality index indicates whether a signal meets quality standards, having successfully undergone these two assessment stages. A 200 Hz downsampling rate was applied. For downsampled signals, standardizing sample rates is essential to ensure uniformity across all signals, ensuring a consistent processing pipeline filtration regardless of its acquisition sample rates. If this index exceeded a minimal threshold, a binary classifier trained using 21,000 manually classified pulses from five experienced professionals was employed to assess the final pulse validity. Finally, manual data filtering was conducted by three experienced professionals (GNO, DC, and GF). They applied the following criteria to deem data valid for analysis: out of the 5-minute ICC collected data, at least one minute of valid monitoring was required; minutes with excessive noise due to volunteer movement were disregarded, as reported through observation field or visual inspection. Additionally, pulse morphology was checked for accuracy. An illustration of ICP pulse morphology and parameters obtained by the brain4care system is presented in Fig. 1.

### Sample size calculation

Due to the exploratory nature of this study and the limited resources, a sample size of convenience was considered.

### Statistical methods

A descriptive statistical analysis was employed in the study. For the variables collected, once it was determined that the data were not normally distributed, the data were presented as the median with the interquartile range and as a percentage for the categorical variables. Age was stratified into 4 groups: children (≤ 7 years); young adults (18 ≤ age ≤ 44 years); middle-aged adults (45 ≤ age ≤ 64 years); older adults (≥ 65 years). The associations between the P2/P1 ratio, TTP, and the clinical variables were evaluated using the Mann-Whitney U test, based on the non-normally distributed data. Multiple linear regression was utilized to identify variables most strongly associated with the P2/P1 ratio and TTP. The level of statistical significance was set at *p* < 0.05. For data analysis and graphical representations of the data, R software was used (version 4.3.2).

## Results

**Fig. 2:**
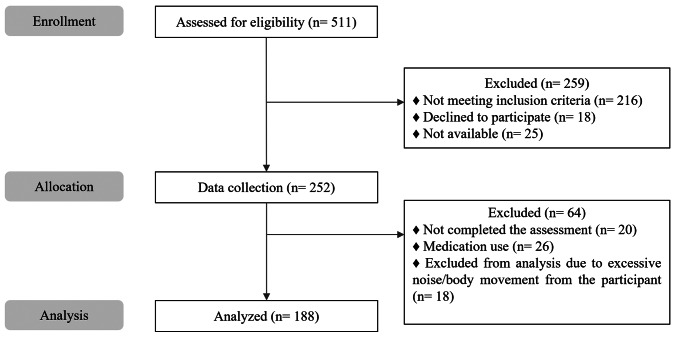
Flow diagram of participants in accordance with CONSORT [16]

From December 2020 to March 2023, 511 eligible participants were identified. After exclusion of 259 participants, 252 were allocated to complete the assessments, and data were analyzed from 188 participants. A detailed participant flow according to CONSORT is shown in Fig. 2.

### Participants characteristics and data distribution

Demographic data from the analyzed participants are presented in Table 1. The age group distribution is presented in Table 2. The participants’ data related to ICC - P2/P1 ratio and TTP - were presented in different age categories. From the health history assessment, some volunteers presented hypertension, diabetes, cardiac or pulmonary condition, or obesity as a health condition. Although the volunteers made a self-report of anxiety, they were not clinically diagnosed. The symptoms reported were related to feelings of worry or tension.

**Table 1:** Characteristics of study participants

		Median and Interquartile range (1º − 3º)
Age		
		41 (29–51)
		***N*** **(%)**
		188 (100%)
Sex		
	Male	104 (55.32%)
	Female	84 (44.68%)
Ethnicity		
	Caucasian	119 (63.30%)
	Black	3 (1.60%)
	Mixed race / brown	58 (30.85%)
	Hispanic	5 (2.66%)
	Asian	3 (1.60%)
Body mass index classification		
	Underweight	4 (2.13%)
	Normal weight	88 (46.81%)
	Overweight	75 (39.89%)
	Obese I	18 (9.57%)
	Obese II	3 (1.60%)
Waist-hip ratio classification		
	Low	58 (30.85%)
	Moderate	56 (29.79%)
	High	74 (39.36%)
Alcohol ingestion?		
	No	154 (81.91%)
	Yes	34 (18.09%)
Smoking?		
	No	178 (94.68%)
	Yes	10 (5.32%)
Sleep disorders?		
	No	144 (76.60%)
	Yes	44 (23.40%)
Physical activity?		
	No	43 (22.87%)
	Yes	145 (77.13%)
Hypertension?		
	No	183 (97.34%)
	Yes	5 (2.66%)
Diabetes?		
	No	185 (98.40%)
	Yes	3 (2.66%)
Anxiety?		
	No	145 (77.13%)
	Yes	43 (22.87%)
History of migraine?		
	No	163 (86.70%)
	Yes	25 (13.30%)
Cardiac or pulmonary condition?		
	No	180 (95.74%)
	Yes	8 (4.26%)
Type of cardiac or pulmonary condition		
	Asthma	7 (87.50%)
	Arrhythmia	1 (12.50%)

**Table 2:** Age group distribution

Sex	Age group (*N*)			
	1 (≤ 17 years)	2 (18 ≤ age ≤ 44 years)	3 (45 ≤ age ≤ 64 years)	4 (≥ 65 years)
Female	5	49	26	4
Male	7	51	43	3

N: sample size

#### Intracranial compliance data

**Fig. 3:**
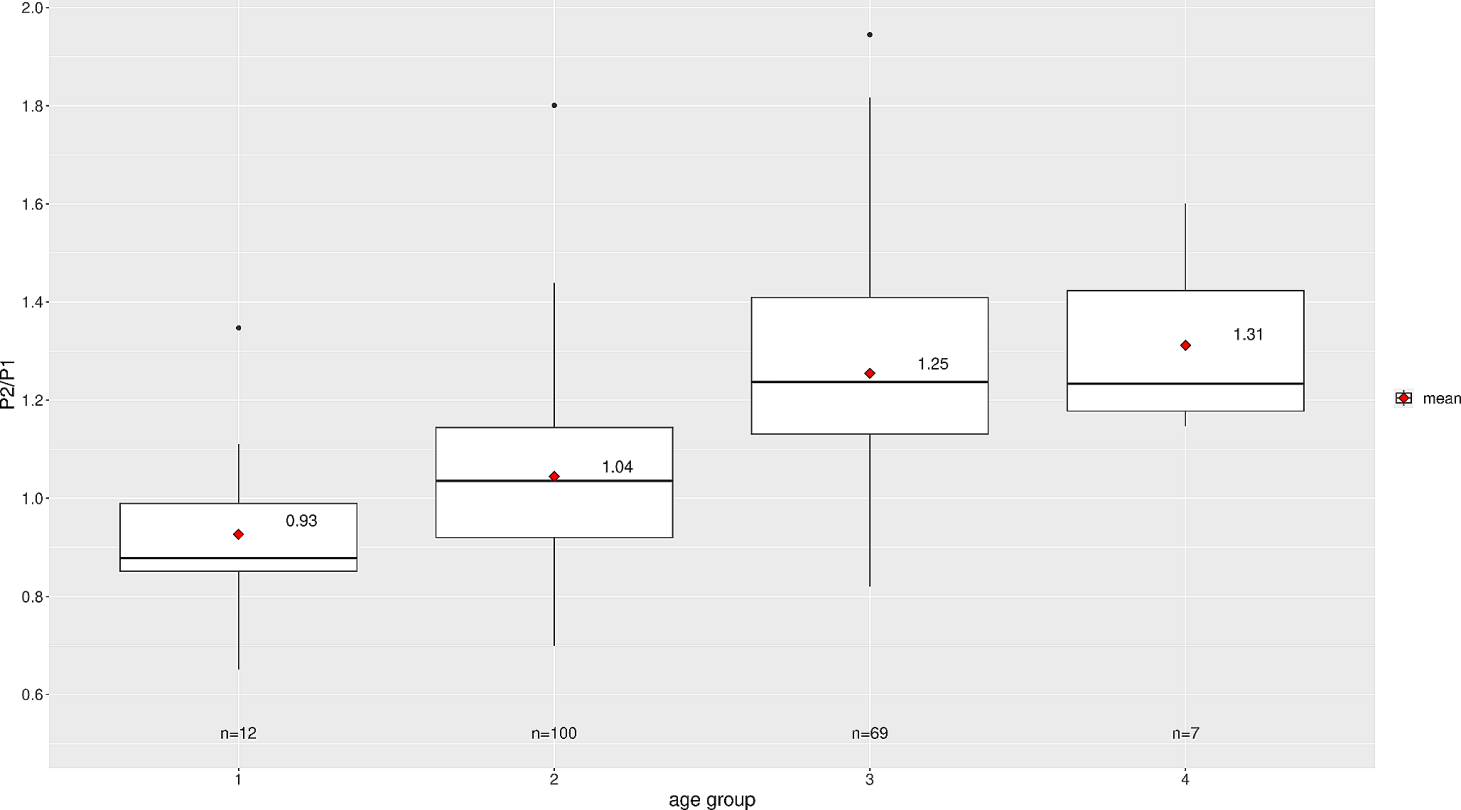
P2/P1 ratio distribution by age group

**Fig. 4:**
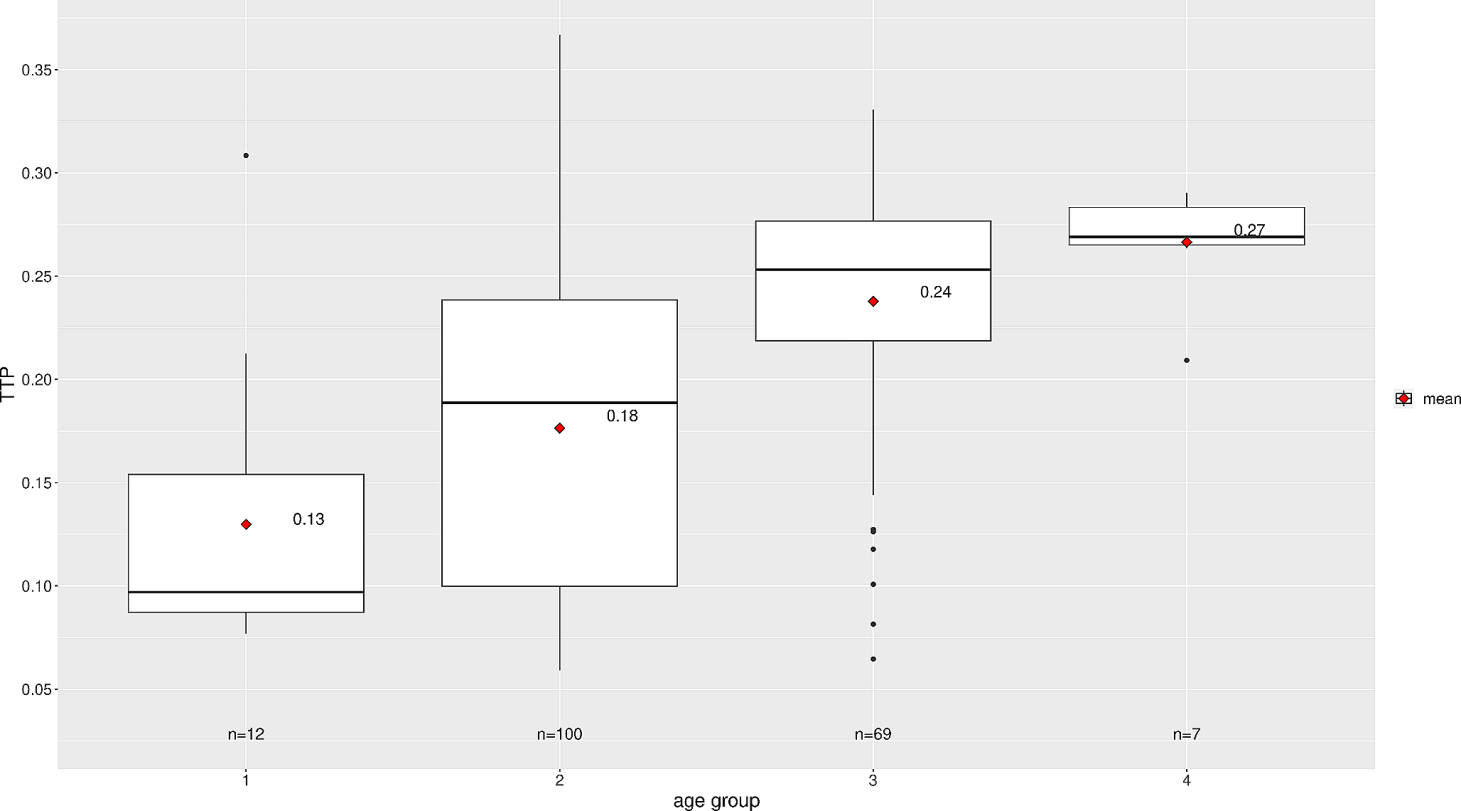
TTP distribution by age group

**Fig. 5:**
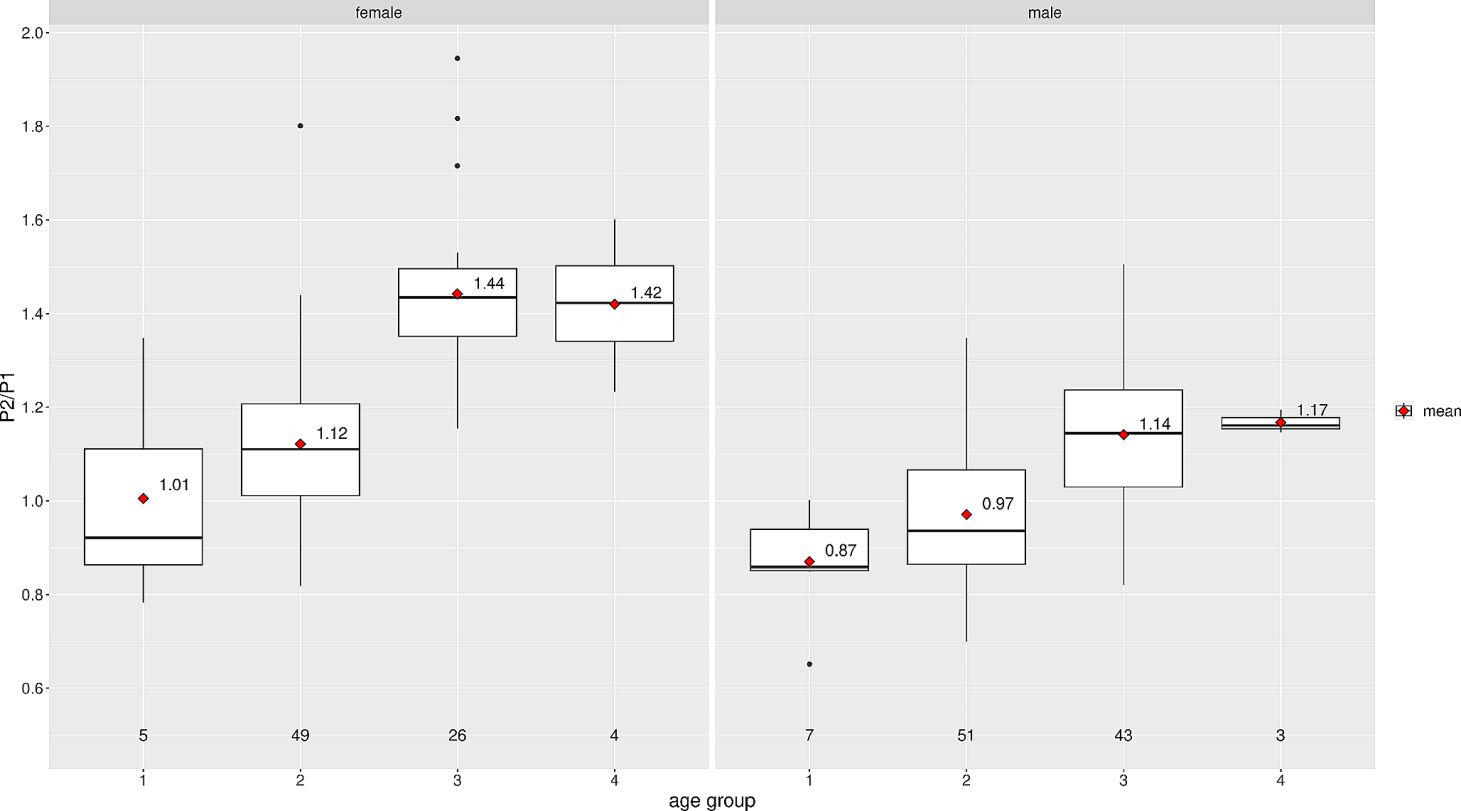
P2/P1 ratio distribution by sex and age group

**Fig. 6:**
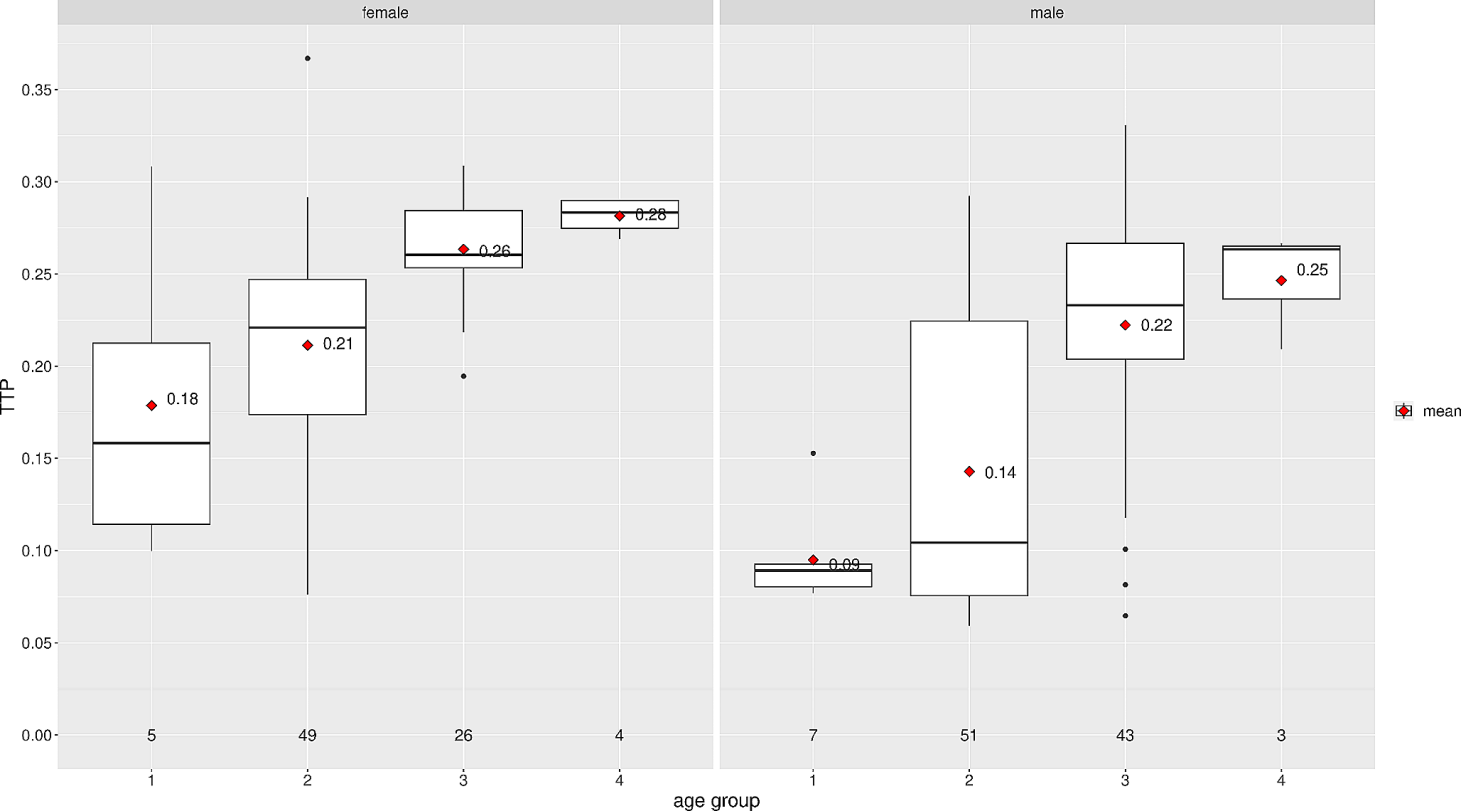
TTP distribution by sex and age group

The ICP pulse waveform parameters, including the P2/P1 ratio and TTP, were established as reference values in the horizontal supine position at 0 degrees. Across all groups, the reference values were found to be consistent across age distribution, as shown in Table 3; Fig. 3 for the P2/P1 ratio and Fig. 4 for TTP. For visualization of parameters segregated by different sexes, data for the P2/P1 ratio and TTP can be observed in Figs. 5 and 6, respectively.

**Table 3:** P2/P1 ratio and TTP presented by age group

Variable	Statistic	Age group				
		Age groups merged	1 Children (≤ 17 years)	2 Young adults (18 ≤ age ≤ 44 years)	3 Middle-aged adults (45 ≤ age ≤ 64 years)	4 Older adults (≥ 65 years)
	*N*	188	12	100	69	7
P2/P1 ratio	Median	1.12	0.88	1.04	1.24	1.23
	Interquartile range (1º − 3º)	0.95–1.25	0.85–0.99	0.92–1.14	1.13–1.41	1.18–1.42
TTP	Median	0.22	0.10	0.18	0.24	0.27
	Interquartile range (1º − 3º)	0.12–0.26	0.09–0.15	0.10–0.24	0.22–0.28	0.27–0.28

N: sample size; 1º: first quartile; 3º: third quartile

The central horizontal lines represent the median of the sample, the upper hinge of the box represents the third quartile and the lower hinge represents the first quartile. The upper whisker extends from the hinge to the largest value no further than 1.5 * IQR (interquartile range, difference between third and first quartile) from the hinge. The lower whisker extends from the hinge to the smallest value at most 1.5 * IQR of the hinge. Data beyond the end of the whiskers are considered outliers and are plotted individually. Values displayed in the boxplot reflect the mean.

The central horizontal lines represent the median of the sample, the upper hinge of the box represents the third quartile and the lower hinge represents the first quartile. The upper whisker extends from the hinge to the largest value no further than 1.5 * IQR (interquartile range, difference between third and first quartile) from the hinge. The lower whisker extends from the hinge to the smallest value at most 1.5 * IQR of the hinge. Data beyond the end of the whiskers are considered outliers and are plotted individually. Values displayed in the boxplot reflect the mean.

The central horizontal lines represent the median of the sample, the upper hinge of the box represents the third quartile and the lower hinge represents the first quartile. The upper whisker extends from the hinge to the largest value no further than 1.5 * IQR (interquartile range, difference between third and first quartile) from the hinge. The lower whisker extends from the hinge to the smallest value at most 1.5 * IQR of the hinge. Data beyond the end of the whiskers are considered outliers and are plotted individually. Values displayed in the boxplot reflect the mean.

The central horizontal lines represent the median of the sample, the upper hinge of the box represents the third quartile and the lower hinge represents the first quartile. The upper whisker extends from the hinge to the largest value no further than 1.5 * IQR (interquartile range, difference between third and first quartile) from the hinge. The lower whisker extends from the hinge to the smallest value at most 1.5 * IQR of the hinge. Data beyond the end of the whiskers are considered outliers and are plotted individually. Values displayed in the boxplot reflect the mean.

#### Sex-related reference values for P2/P1 ratio and TTP

A difference between women and men was observed in the data for the P2/P1 ratio. In the women’s group, the middle-aged adult group and the old adult group showed an increase in the P2/P1 ratio with a median of 1.43 (IQR: 1.35–1.50) and 1.42 (IQR: 1.34–1.50), respectively. The children group showed the lowest values for P2/P1 ratio at 0.92 (IQR: 0.86–1.11), while the young adults group showed a median P2/P1 ratio of 1.11 (IQR: 1.01–1.21). In men, the median P2/P1 ratio was 1.14 (IQR: 1.14–1.24) in the middle-aged adult group and 1.16 (IQR: 1.16–1.18) in the old adult group. Further details are shown in Figs. 5 and 6. The same behavior can be observed with TTP, where the increase begins in middle-aged adult groups in both sexes.

#### Age-related reference values for P2/P1 ratio and TTP

Reference values for the P2/P1 ratio and TTP displayed an increasing trend starting from the middle-aged adults group, with a median of 1.24 (IQR: 1.13–1.41) and 0.24 (IQR: 0.22–0.28), respectively. The children group had the lowest median P2/P1 ratio of 0.88 (IQR: 0.85–0.99).

#### Characterization variables affecting P2/P1 ratio and TTP

The results indicated that variables such as waist-to-hip ratio (*p* = 0.01) were associated with higher P2/P1 ratio and TTP, respectively. Furthermore, body mass index classification (*p* = 0.01) was associated with higher TTP. However, the remaining variables were not associated with higher P2/P1 ratio or TTP. The data are presented in Table 4.

**Table 4:** Characterization variables data analysis for P2/P1 ratio and TTP

		P2/P1 ratio median (Interquartile range: 1º − 3º)	*p*-value	TTP median (Interquartile range: 1º − 3º)	*p*-value
Sex					
	Male	1.02 (0.90–1.16)	< 0.001*	0.19 (0.09–0.25)	< 0.001*
	Female	1.21 (1.06–1.39)		0.24 (0.20–0.27)	
Body mass index classification					
	C1	1.11 (0.94–1.24)	0.07	0.22 (0.12–0.26)	0.01*
	C2	1.15 (1.03–1.41)		0.26 (0.20–0.29)	
Waist-hip ratio classification					
	C1A	1.10 (0.94–1.22)	0.01*	0.21 (0.10–0.25)	0.01*
	C2B	1.18 (1.00–1.38)		0.24 (0.16–0.27)	
Alcohol ingestion?					
	No	1.12 (0.95–1.26)	0.49	0.22 (0.12–0.26)	0.27
	Yes	1.09 (0.98–1.13)		0.23 (0.13–0.28)	
Smoking?					
	No	1.12 (0.97–1.26)	0.17	0.22 (0.13–0.26)	0.25
	Yes	0.99 (0.91–1.12)		0.14 (0.11–0.23)	
Sleep disorders?					
	No	1.12 (0.95–1.24)	0.77	0.22 (0.12–0.37)	0.48
	Yes	1.09 (0.97–1.33)		0.23 (0.15–0.26)	
Physical activity?					
	No	1.07 (0.92–1.23)	0.29	0.17 (0.10–0.27)	0.61
	Yes	1.13 (0.98–1.26)		0.23 (0.14–0.26)	
Hypertension?					
	No	1.12 (0.96–1.25)	0.74	0.22 (0.13–0.26)	0.74
	Yes	1.14 (0.89–1.21)		0.26 (0.09–0.27)	
Diabetes?					
	No	1.12 (0.95–1.24)	0.18	0.22 (0.13–0.26)	0.97
	Yes	1.31 (1.11–1.34)		0.23 (0.16–0.25)	
Symptoms of anxiety?					
	No	1.13 (0.98–1.26)	0.12	0.23 (0.14–0.27)	0.17
	Yes	1.03 (0.92–1.19)		0.17 (0.11–0.25)	
History of migraine?					
	No	1.13 (0.96–1.26)	0.35	0.23 (0.12–0.26)	0.47
	Yes	1.05 (0.94–1.21)		0.17 (0.13–0.26)	
Cardiac or pulmonary condition?					
	No	1.12 (0.96–1.26)	0.25	0.22 (0.13–0.26)	0.83
	Yes	0.99 (0.93–1.13)		0.14 (0.11–0.28)	

CI: confidence interval; C1: category 1 - underweight, normal weight and overweight; C2: category 2 - obese I and II; C1A: category 1 A - low and moderate risk; C2B: category 2B - high risk. * significance level < 0.05

#### Multivariate analysis

In the multivariate analysis, age and sex remained significantly associated with the P2/P1 ratio and TTP. The *p*-values for each variable were < 0.001. Details are illustrated in Table 5. For both the P2/P1 ratio and TTP, men exhibit lower values compared to women. As age increases, there is a relative rise in both P2/P1 and TTP.

**Table 5:** Multivariate analysis data

Coefficients	P2/P1 ratio			TTP		
	estimate	standard error	*p*-value	estimate	standard error	*p*-value
Intercept	0.947	0.045	< 0.001 *	0.137	0.017	< 0.001 *
Sex male	-0.214	0.028	< 0.001 *	-0.062	0.011	< 0.001 *
Age	0.008	0.001	< 0.001 *	0.002	0.0004	< 0.001 *
BMI_class C2	0.047	0.043	0.270	0.020	0.017	0.235
WHR_class C2B	-0.023	0.029	0.443	-0.007	0.011	0.537
Physical_activity	-0.043	0.032	0.181	-0.002	0.012	0.853
Hypertension	0.018	0.081	0.823	0.016	0.031	0.610
Diabetes	-0.032	0.101	0.753	-0.036	0.039	0.352
Cardiac or pulmonary condition	0.020	0.066	0.763	0.023	0.025	0.367

BMI_class C2: body mass index category 2 - obese I and II; WHR_class C2B: waist-hip ratio category 2B - high risk; * significance level < 0.05

## Discussion

The results of our study suggest that age and sex significantly impact P2/P1 ratio and TTP values. Hence, the necessity for age and sex-adjusted reference values is underscored. This study monitored the characteristics of the ICP waveform and established reference values for the brain4care system parameters, specifically the P2/P1 ratio and TTP, in healthy subjects outside the neurocritical care context. The findings reveal that age and sex exert an influence on the P2/P1 ratio and TTP among healthy individuals. Previous literature reports have demonstrated P2/P1 values exceeding 1.20 and TTP values surpassing 0.25 in neurocritical care or health conditions, indicating potential alterations in intracranial compliance [6–10]. Similarly, when examining data independent of age and sex influences, a median P2/P1 ratio of 1.12 and TTP values of 0.22 were observed in our study. However, significant differences in the P2/P1 ratio were found in women, especially from middle-aged onwards, raising pivotal questions about possible reductions in intracranial compliance with aging and factors influencing this reduction in females.

The existing literature indicates that cerebral volume declines continuously with age, particularly after the age of 40 [17]. Additionally, there is an increase in intracranial cerebrospinal fluid volume with aging, which is related to the reduction of brain volume [18]. Furthermore, there are estimated reductions in brain rigidity by approximately 0.3–1.0% annually [19–21]. Nevertheless, female brains are on average 9% stiffer than male brains, indicating that arterial stiffness may be a significant factor in compliance reduction with age [22–24].

Previous research by Santos et al. and Fantin et al. highlighted that the augmentation index (AI), an indirect measure of arterial stiffness, increases with age and is higher in women [21, 25]. This is measured by the ratio of augmentation pressure (PAum) to pulse pressure (PP) times 100. Cardiovascular disease development is more prevalent in men, but AI and PAum are higher in women, possibly due to shorter stature and closer physical proximity between the heart and reflection sites. A recent meta-analysis by Lu et al. with over 500,000 individuals found that arterial pulse wave velocity (PWV), an indirect measure of arterial wall stiffness, increases with age and is associated with a higher incidence of cerebrovascular events in Asian populations [21, 25, 26].

Furthermore, sex hormones appear to play a role in arterial stiffness, and female sex hormones have a pathological role in idiopathic intracranial hypertension [27, 28]. During menopause, the loss of sex hormones seems to be associated with an increased risk of cardiovascular disease [29]. The changes in sex hormones experienced by women could be another explanation why P2/P1 ratio and TTP are higher when compared to men.

The exact origin of peaks in the ICP pulse waveform remains elusive, as noted by Czosnyka and Czosnyka [30]. However, it is widely accepted that the global shape of this waveform results from the interaction of arterial and venous factors, along with intracranial volume-pressure dynamics. The initial peak, P1, aligns with the systolic apex of arterial pressure, reflecting arterial pulse propagation and immediate arterial wall expansion. Subsequent peaks, P2 and P3, seem linked to fluctuations in cerebral blood volume and intrinsic compensatory mechanisms. Thus, increased arterial rigidity could lead to a reduction in the P1 peak and a consequent increase in the P2/P1 ratio with age [30].

Gholampour and colleagues emphasize that the assessment of intracranial compliance presents significant challenges, primarily due to the nature of the direct measurement to be obtained through invasive procedures [31]. Nevertheless, they demonstrate that noninvasive techniques have been employed to estimate ICC without the capacity to directly measure or calculate ICC values. One of the most frequently employed methodologies for forecasting alterations in intracranial compliance is the curve generated by the pulse wave of intracranial pressure, which is transmitted from the heart through the cerebral vessels to the brain [31]. The ICP pulse waveform peaks can be utilized to provide insight into alterations in intracranial compliance. When P2 is greater than P1, it can be inferred that there has been a change in intracranial compliance, as indicated by the P2/P1 ratio [32].

The use of the brain4care system provides a novel, non-invasive approach to evaluating ICP dynamics, which could significantly reduce the risks associated with traditional invasive methods. The potential for this technology to serve as a predictive tool is particularly promising, as it offers a means for early detection of neurological conditions, thereby enhancing diagnostic accuracy for patients with conditions affecting ICP. The principle of brain4care involves measuring skull deformation to capture pulsatile cranial expansion signals. The sensor captures the ICP pulse waveform as analog signals, which are then transformed into a digital signal capable of generating parameters such as the P2/P1 ratio and TTP [15].

The non-invasive nature of the measurement and the ease of use make brain4care a practical tool for both clinical and research settings. These findings pave the way for future studies to validate the P2/P1 ratio as a reliable biomarker for the early detection of conditions associated with alterations in ICC.

### Limitations

The study population, while diverse in age, may not be representative of the general population, as all participants were volunteers and may have a specific health status that is not reflective of broader demographics. Future longitudinal studies are needed to track changes in ICP parameters over time and to expand the demographic diversity of the study population. Additionally, increasing the sample size for each age category would be beneficial. In relation to sex, examinations to map sex hormones could aid in better interpreting their influence.

A potential source of variability in this study could be related to how the sensor was handled and positioned by the operator. Andrade and colleagues provided data from bench tests describing the precision of the sensor, indicating that sensor handling and positioning contributed to 64.08% of the observed overall variability [15]. The supplementary materials include an illustration of the repeatability (see supplementary Tables 1 and supplementary Fig. 1) and stability (see supplementary Tables 2 and supplementary Fig. 2) parameters derived from data collected from a volunteer. It is recommended that a monitoring session commence only when the sensor is properly adjusted and the signal is stable on the monitor.

## Conclusion

Our findings indicate that the P2/P1 ratio and TTP are influenced by age and sex in healthy subjects. For both the P2/P1 ratio and TTP, men exhibit lower values compared to women. As age increases, there is a relative rise in both P2/P1 and TTP. These preliminary data, focusing on characterizing the morphology of the ICP waveform outside the neurocritical care context and utilizing parameters such as P2/P1 and TTP ratio, provide insights for future studies. Such studies could involve larger sample sizes and diverse age groups, aiming to establish reference points for comparisons in scenarios involving established or acquired health conditions. Furthermore, these results endorse the utilization of ICP waveform parameters to distinguish between normal and abnormal ICC and ICP.

## Electronic supplementary material

Below is the link to the electronic supplementary material.

Supplementary Material 1

## Data Availability

Data is provided within the manuscript.
