# Supplementary material for: Characterization of intracranial compliance in healthy subjects using a noninvasive method - results from a multicenter prospective observational study
Source: J Clin Monit Comput. 2024 Jul 20;38(6):1249–61. doi: 10.1007/s10877-024-01191-w (PMC11604689; doi:10.1007/s10877-024-01191-w)

Supplementary table 1. Data from Brain4care acquisitions on a single individual- repeatability

| Minute | Repetition 1 | | Repetition 2 | | Repetition 3 | | Repetition 4 | | Repetition 5 | |
| --- | --- | --- | --- | --- | --- | --- | --- | --- | --- | --- |
|  | P2/P1 ratio | TTP | P2/P1 ratio | TTP | P2/P1 ratio | TTP | P2/P1 ratio | TTP | P2/P1 ratio | TTP |
| 1 | 1.01 | 0.12 | 0.97 | 0.10 | 0.91 | 0.09 | 0.92 | 0.07 | 0.94 | 0.10 |
| 2 | 0.98 | 0.11 | 0.95 | 0.10 | 0.89 | 0.08 | 0.94 | 0.09 | 0.88 | 0.09 |
| 3 | 0.96 | 0.10 | 0.94 | 0.10 | 0.90 | 0.08 | 0.98 | 0.11 | 0.88 | 0.09 |
| 4 | 0.96 | 0.10 | 0.94 | 0.10 | 0.88 | 0.08 | 0.99 | 0.11 | 0.90 | 0.09 |
| 5 | 0.96 | 0.10 | 0.94 | 0.10 | 0.88 | 0.08 | 1.00 | 0.12 | 0.90 | 0.09 |
| Mean | 0.97 | 0.11 | 0.95 | 0.10 | 0.89 | 0.08 | 0.97 | 0.10 | 0.90 | 0.09 |
| Standard deviation | 0.02 | 0.01 | 0.01 | 0.00 | 0.01 | 0.00 | 0.03 | 0.02 | 0.02 | 0.00 |

Supplementary table 2. Data from Brain4care acquisitions on a single individual - stability

| Minute | 1 | 2 | 3 | 4 | 5 | 6 | 7 | 8 | 9 | 10 |
| --- | --- | --- | --- | --- | --- | --- | --- | --- | --- | --- |
| P2/P1 | 1.0 | 0.99 | 0.97 | 0.95 | 0.94 | 0.93 | 0.94 | 0.92 | 0.91 | 0.91 |
| TTP | 0.11 | 0.11 | 0.11 | 0.11 | 0.11 | 0.11 | 0.11 | 0.10 | 0.10 | 0.09 |
| Minute | 11 | 12 | 13 | 14 | 15 | 16 | 17 | 18 | 19 | 20 |
| P2/P1 | 0.91 | 0.91 | 0.91 | 0.91 | 0.92 | 0.92 | 0.91 | 0.90 | 0.89 | 0.90 |
| TTP | 0.09 | 0.10 | 0.10 | 0.09 | 0.10 | 0.09 | 0.10 | 0.10 | 0.10 | 0.09 |
| Minute | 21 | 22 | 23 | 24 | 25 | 26 | 27 | 28 | 29 | 30 |
| P2/P1 | 0.91 | 0.89 | 0.90 | 0.90 | 0.91 | 0.89 | 0.90 | 0.90 | 0.89 | 0.90 |
| TTP | 0.10 | 0.09 | 0.09 | 0.09 | 0.09 | 0.09 | 0.09 | 0.09 | 0.09 | 0.09 |
| Minute | 31 | 32 | 33 | 34 | 35 | 36 | 37 | 38 | 39 | 40 |
| P2/P1 | 0.90 | 0.88 | 0.89 | 0.89 | 0.90 | 0.91 | 0.90 | 0.91 | 0.91 | 0.90 |
| TTP | 0.09 | 0.09 | 0.09 | 0.09 | 0.09 | 0.09 | 0.09 | 0.09 | 0.09 | 0.09 |
| Minute | 41 | 42 | 43 | 44 | 45 | 46 | 47 | - | - | - |
| P2/P1 | 0.90 | 0.90 | 0.91 | 0.91 | 0.91 | 0.91 | 0.92 | - | - | - |
| TTP | 0.10 | 0.09 | 0.09 | 0.09 | 0.09 | 0.09 | 0.10 | - | - | - |
| Mean: 0.91 | | | | | | | | | | |
| Standard deviation: 0.02 | | | | | | | | | | |

Supplementary figure1. Brain4care reports illustrating measurement repeatability on a single individual.

1. Repetition 01


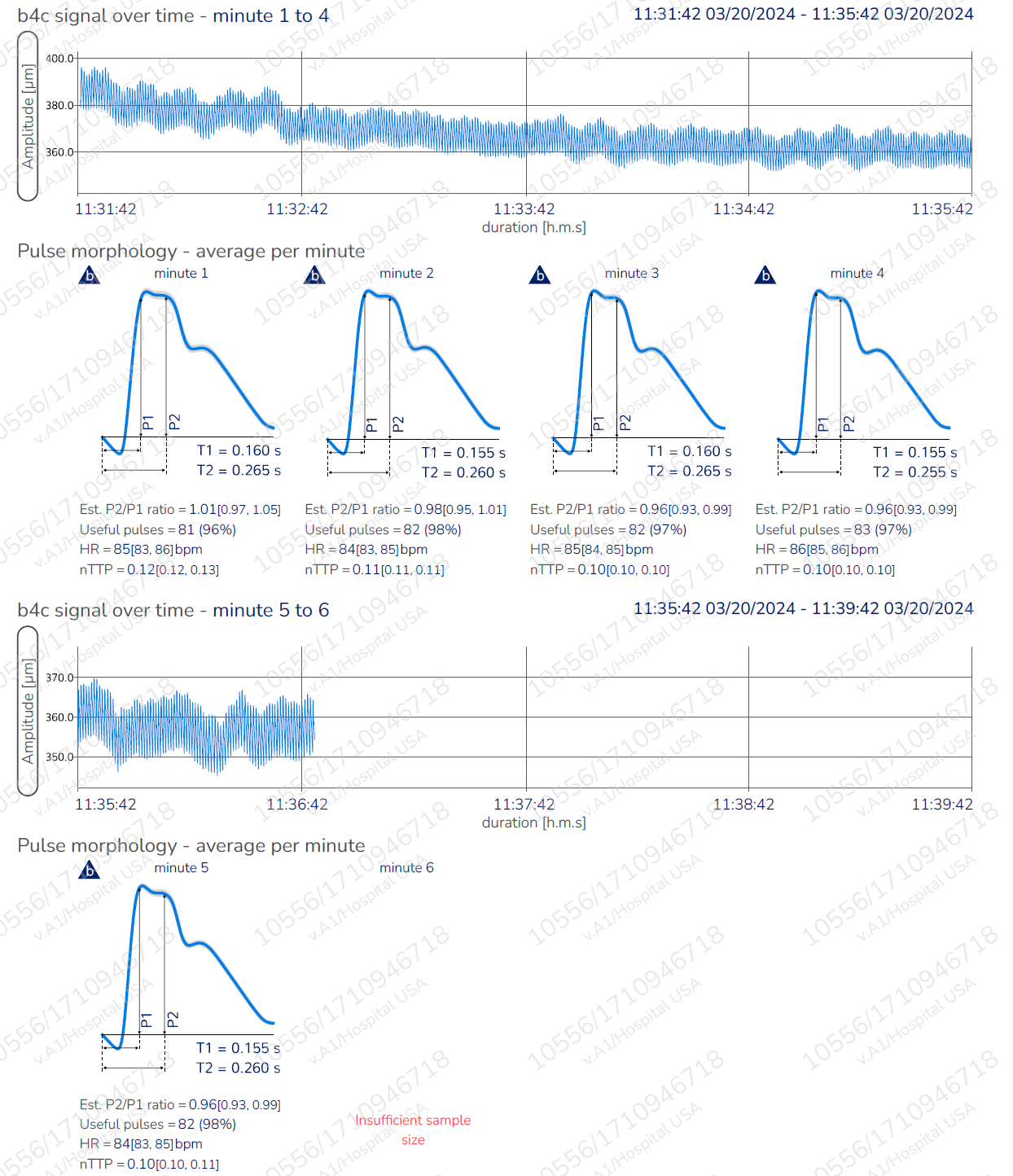


1. Repetition 02


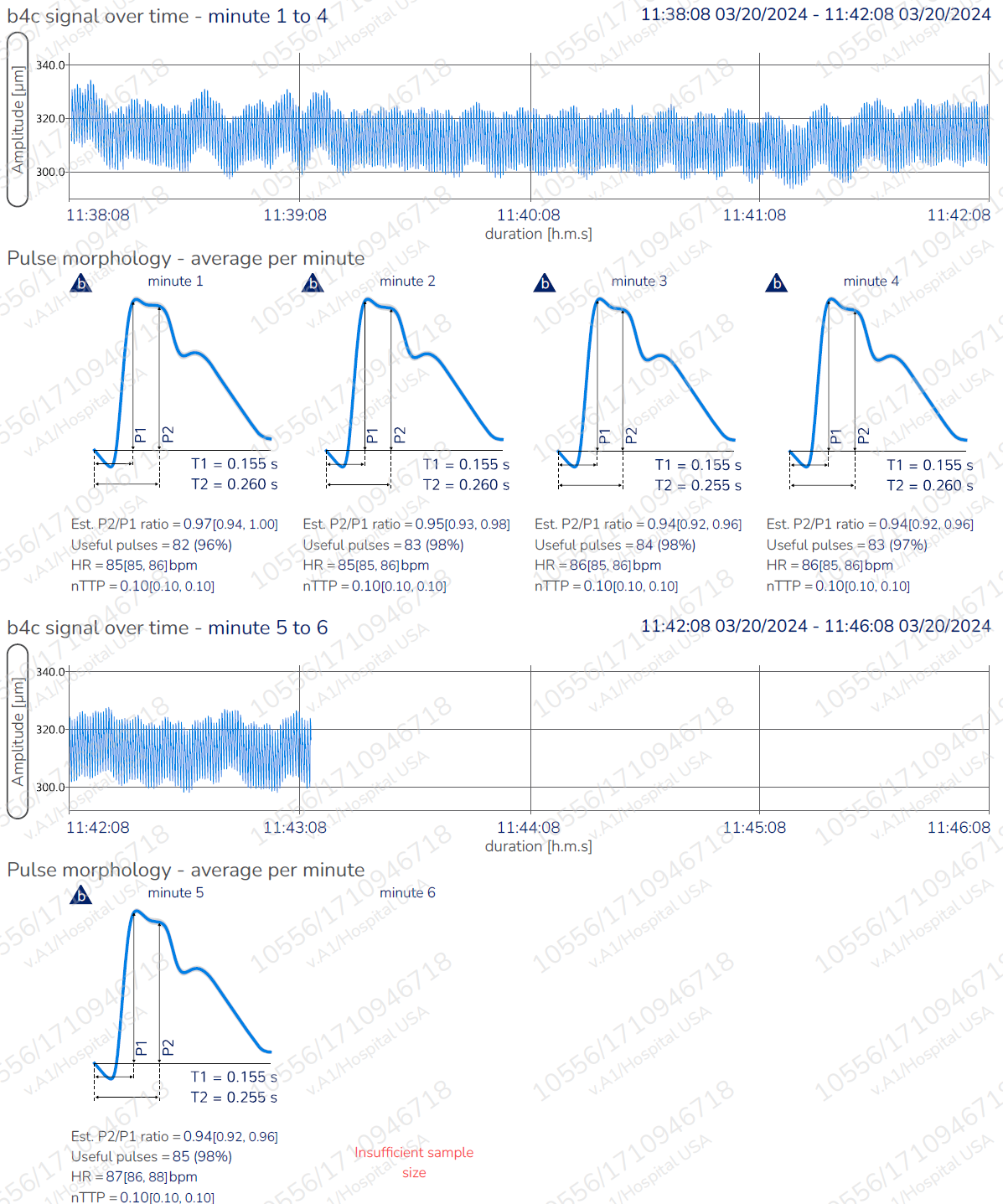


1. Repetition 3


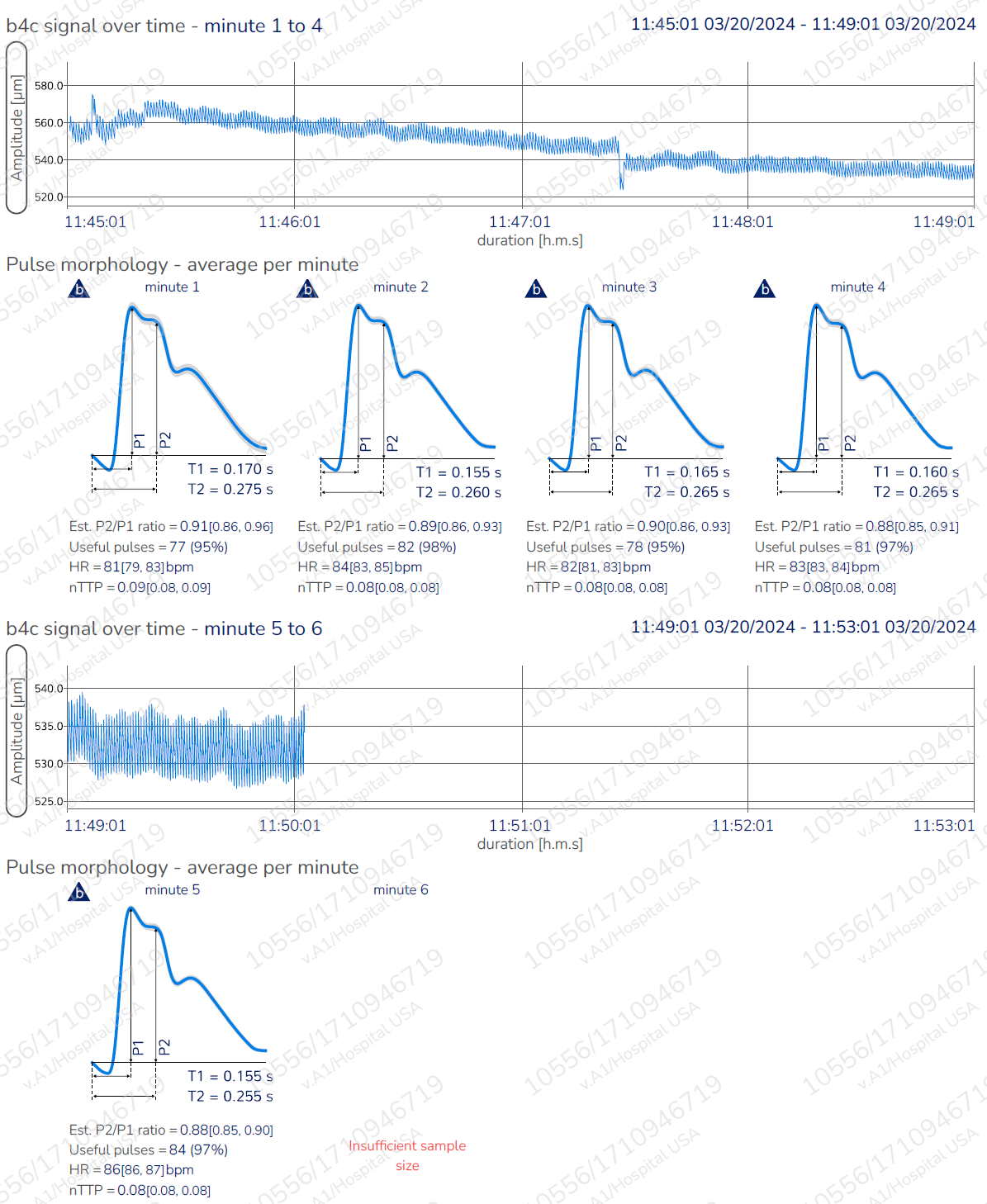


1. Repetition 4


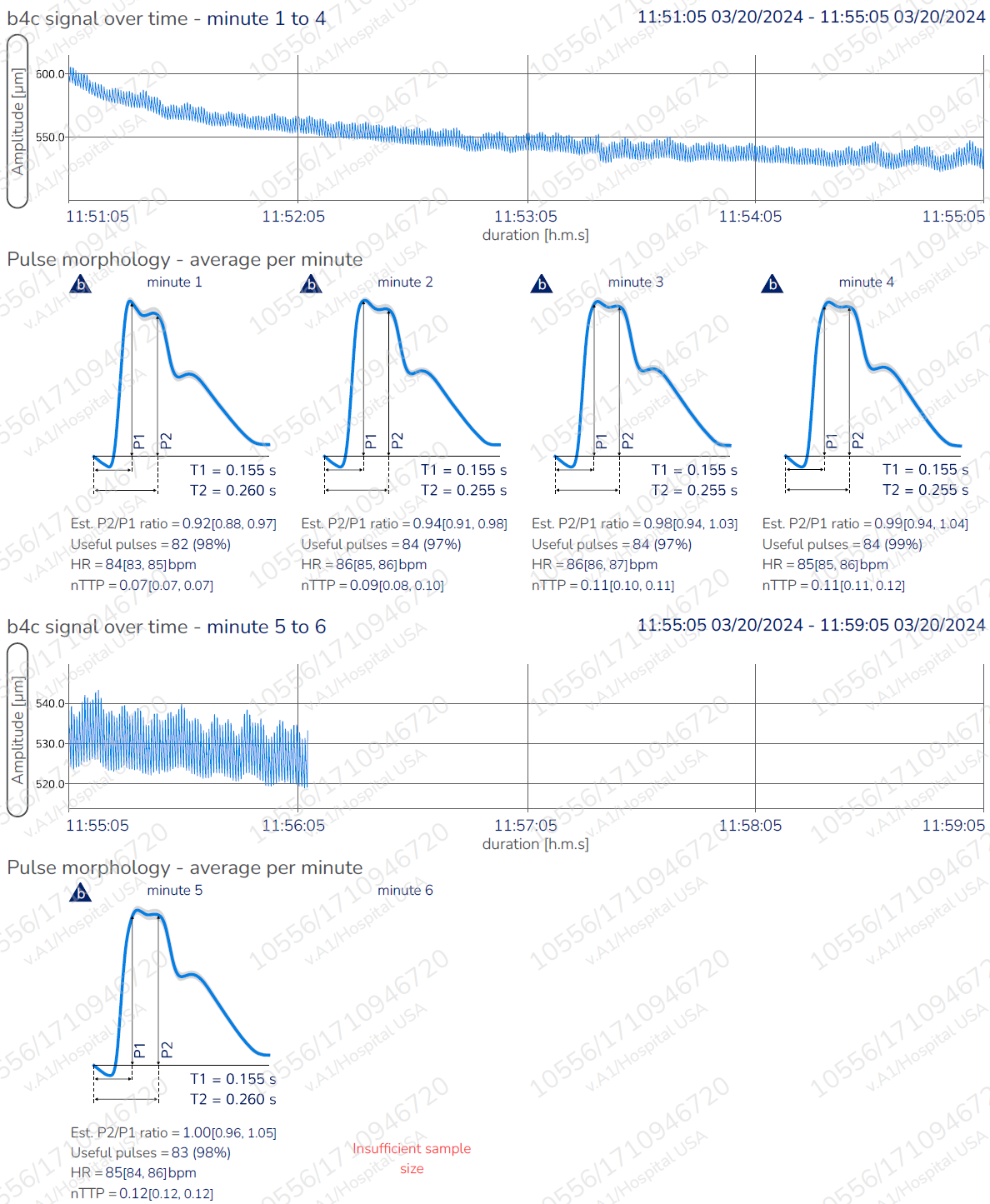


1. Repetition 05


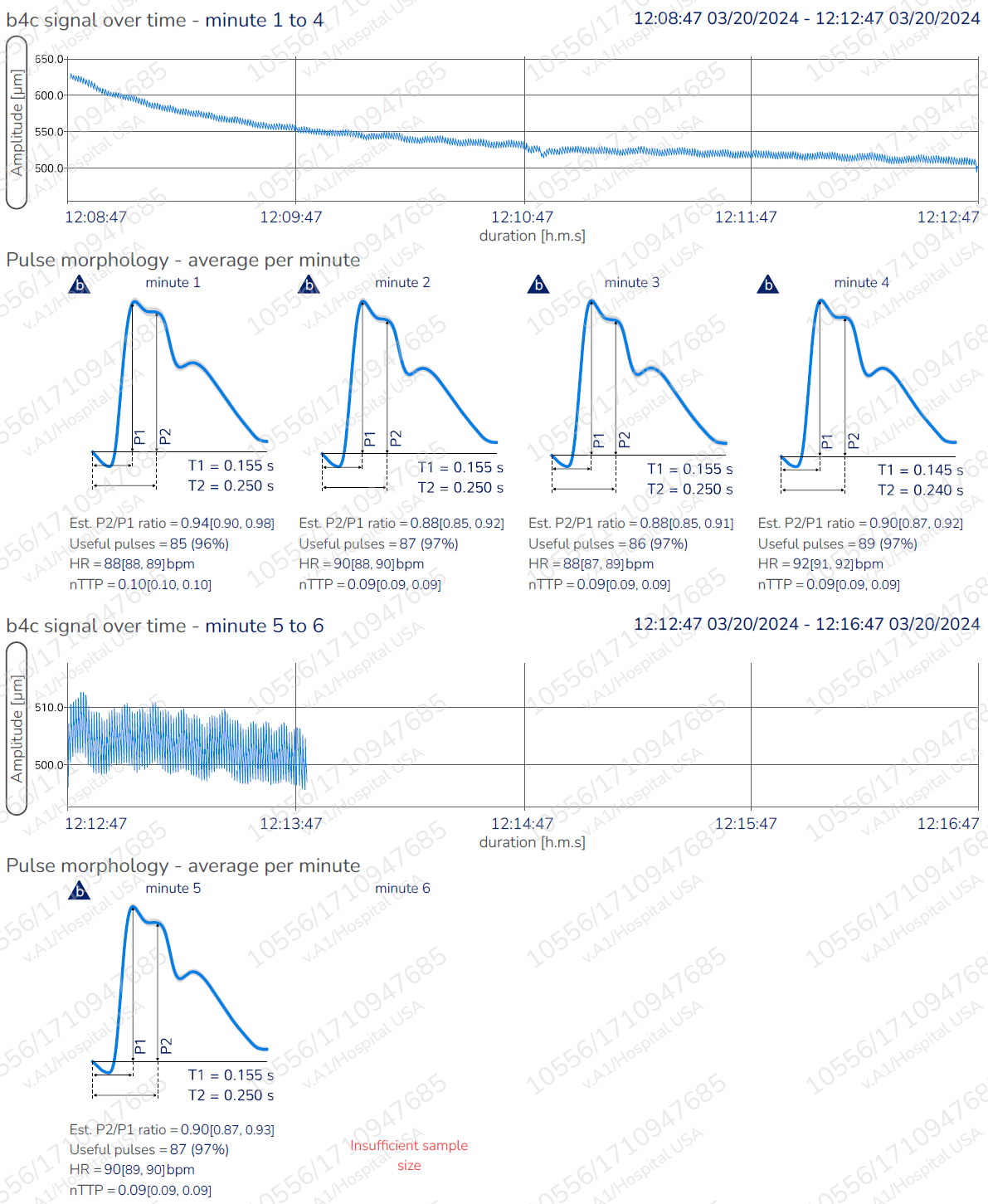


Supplementary figure 2. Brain4care reports illustrating measurement stability over time on a single individual (period of 47 minutes).


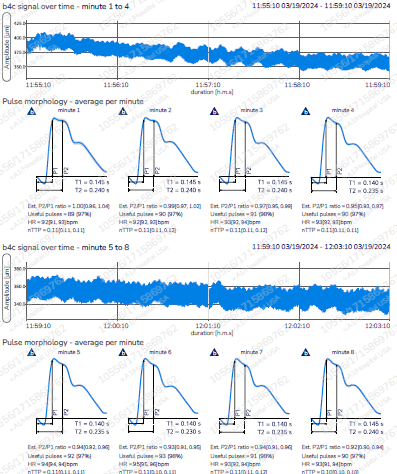

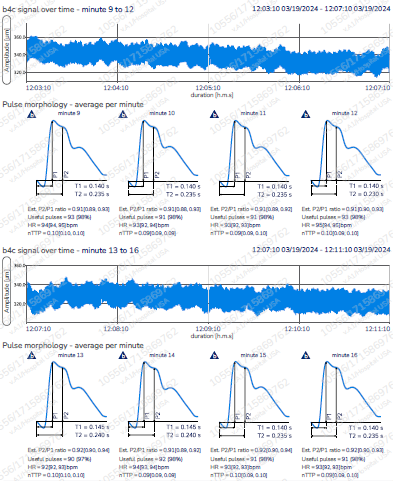


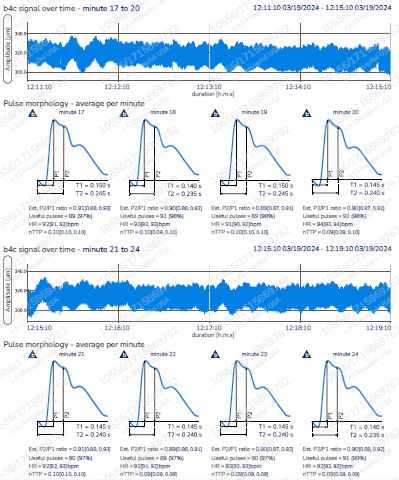

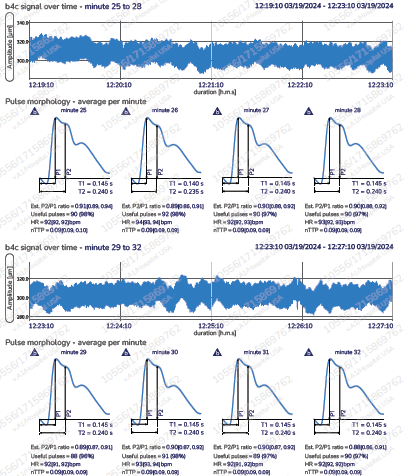

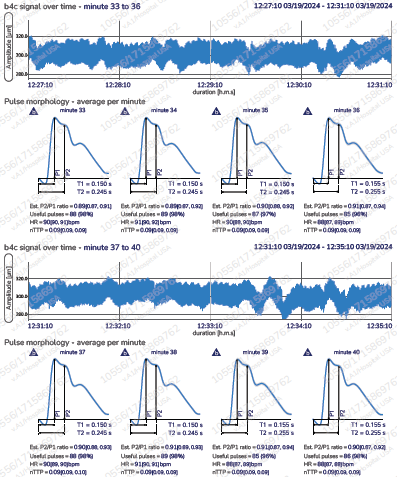

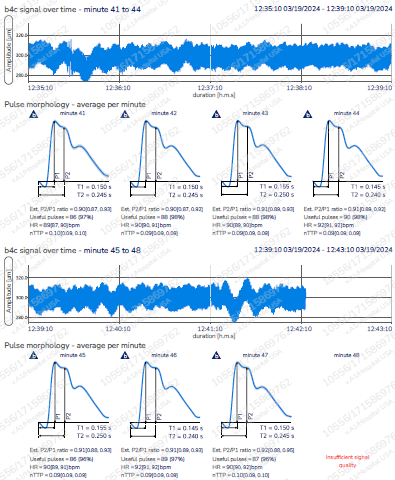

Supplement: Supplementary file 1 — Supplementary Material 1 [file 10877_2024_1191_MOESM1_ESM.docx]
